# Impact of the COVID-19 pandemic and the Russia-Ukraine war on stress and anxiety in students: A systematic review

**DOI:** 10.3389/fpsyt.2022.1081013

**Published:** 2022-11-25

**Authors:** Pierpaolo Limone, Giusi Antonia Toto, Giovanni Messina

**Affiliations:** Learning Science Hub, Department of Human Sciences, University of Foggia, Foggia, Italy

**Keywords:** stress, anxiety, war, COVID-19, pandemic

## Abstract

**Background:**

The outbreak of the COVID-19 pandemic at the end of 2019 and the Russian-Ukrainian war in February 2022 created restrictions and uncertainties that affected the general population’s mental health. One of the affected groups was students. This systematic review summarizes the current literature on the prevalence, outcomes, and interventions for stress and anxiety among university, college, graduate, or postsecondary populations.

**Methods:**

A systematic literature search was performed on PubMed, Web of Science, Scopus, Embase, Cochrane Library, CINAHL, PsychInfo, and ProQuest, following PRISMA guidelines. Specific inclusion and exclusion criteria were applied, and screening was performed to identify the definitive studies.

**Results:**

The prevalence of anxiety was relatively high, ranging from 88.9 to 13.63%, and the prevalence of stress ranged from 56 to 28.14%. The predictors of stress and anxiety included young age, gender being female, STEM course, loneliness, low academic level in school, urban lockdown, confinement, having a preexisting disease, having relatives or friends infected with COVID-19, and proximity to a COVID-19 zone. The predictors of stress included gender being female, living with family, living in a household with many people, being confined rather than having the freedom to relocate, proximity to confirmed cases of COVID-19, lack of access to materials on COVID-19, preexisting mental disorders, and lack of knowledge on the preventable nature of COVID-19. The sources of anxiety among the university students identified in the study included academics, postponement of graduation, cancelation or disruption of planned events, inability to achieve goals, and finances. In addition, the students used trauma-focused, forward-focused, task-oriented, emotion-oriented, and avoidance-oriented coping strategies.

**Conclusion:**

The included studies showed that stress and anxiety increased during the pandemic and the war, with gender and uncertainty playing a critical role. The studies provide insights into the widespread use of problem-focused and task-focused coping strategies despite their impact on increasing stress and anxiety.

## Introduction

A crisis is a period of psychological and physical instability resulting from a dangerous event or situation (1). Crises are unexpected and unforeseen phenomenon and include severe or threatening illness or injury, unexpected death, acts of war or violence, natural or manufactured disasters, and epidemics. During the last two years, two important crises have followed one other in close succession. On the one hand, in December 2019, the first few cases of a novel virus, later referred to as Coronavirus disease 2019 (COVID-19), were first reported in the Wuhan region of China. The declaration of the COVID-19 outbreak as a public health emergency on March 11, 2020, by the World Health Organization led to the disease being recognized as a global pandemic, setting out a series of policy changes and adjustments among individuals and societies (2). Besides illness and death, the global pandemic led to lockdowns, closure of public and non-essential services, loss of employment and income, and the restriction of social movement. Alternative modes of operating and coping were developed to ensure compliance with the spread of the disease. On the other hand, on February 24, 2022, the Russian-Ukrainian War (RUW) began leading to widespread devastation, with approximately 6.7 million refugees fleeing their countries within three months (3). The public health concerns associated with war have been identified to generate significant negative outcomes on the well-being of refugees and the survivors of the war. The effect is also felt in communities close to or supporting either side of the warring countries. While the war likely had a psychological impact on the Ukrainians and Russians within the war zones, the people in central Europe and other countries who were ideologically aligned with the war also experienced some effects. Along with the COVID-19 pandemic, the RUW generated stressors and increased anxiety in different areas of the world (4).

Postsecondary students are one of the demographics impacted by the outbreak of the COVID-19 pandemic and the RUW. Interruptions in school calendars; the introduction of virtual learning; changes in communication channels and workloads; infections; and the death of peers, faculty, family, and friends are some COVID-19-related outcomes that increase stress and mental distress (5). Moreover, the work and life changes imposed on higher-education students were compounded by the social restrictions created by lockdowns and future financial and career uncertainties (6–8). The Internally Displaced Persons Mental Health Survey, which was launched in 2014 in Ukraine after the Russian invasion of Crimea, sheds some light on the potential mental health issues associated with war in the region. The study found that the prevalence of depression (22%), post-traumatic stress disorder (21%), and anxiety (18%) were beyond the accepted thresholds (9). Additionally, those experiencing the problems had reduced access to mental health services. In a study of the mental and emotional impact of the RUW on Ukrainian university students and personnel, Kurapov et al. (10) found that 97.8% of respondents had worse psycho-emotional outcomes with higher depression, loneliness, nervousness, and anger. The findings are consistent with that of other war-time studies that found poor mental health outcomes associated with displacement, shortages, death of family and loved ones, financial uncertainty, and loss of employment (11, 12).

According to the American College Health Association, anxiety is one of the most reported mental health disorders in students and significantly impacts their academic performance (13). According to Son et al. (14), approximately 50% of the students reported anxiety as their primary reason for seeking counseling services at Texas A&M University in 2018. The increased openness to reporting mental health disorders among postsecondary students is an encouraging sign that can be leveraged to provide interventions. However, a better understanding of the prevalence, outcomes, and interventions for anxiety among university students is needed to ensure best practices. Adolescents and young adults, indeed, are depicted by the WHO as high-risk groups for mental disorders, including depression, anxiety, and behavioral disorders. Therefore, the (15) comprehensive mental health action plan 2013–2030 insisted on the explicit inclusion of youth mental health within general and priority health policies.

The main aim of this systematic review is to summarize the current literature on the prevalence, and outcomes of stress and anxiety among university, college, graduate, or postsecondary populations. Specifically, the following systematic review examines the anxiety and stress levels among college and university students in the context of the COVID-19 pandemic and the RUW in order to obtain a clear framework of the effects that these two global crises have had on their wellbeing. Results from this review could usefully inform clinicians, university staff and interventions aimed at supporting higher education students in time of crisis.

## Methods

### Study design

The design used in this study is a systematic review. The literature review uses the Preferred Reporting Items for Systematic reviews and Meta-Analyses guidelines.

### Search strategy

For the study, electronic libraries and databases were used to search and extract articles. This included PubMed, Web of Science, Scopus, Embase, Cochrane Library, CINAHL, PsychInfo, and ProQuest.

**FIGURE 1:**
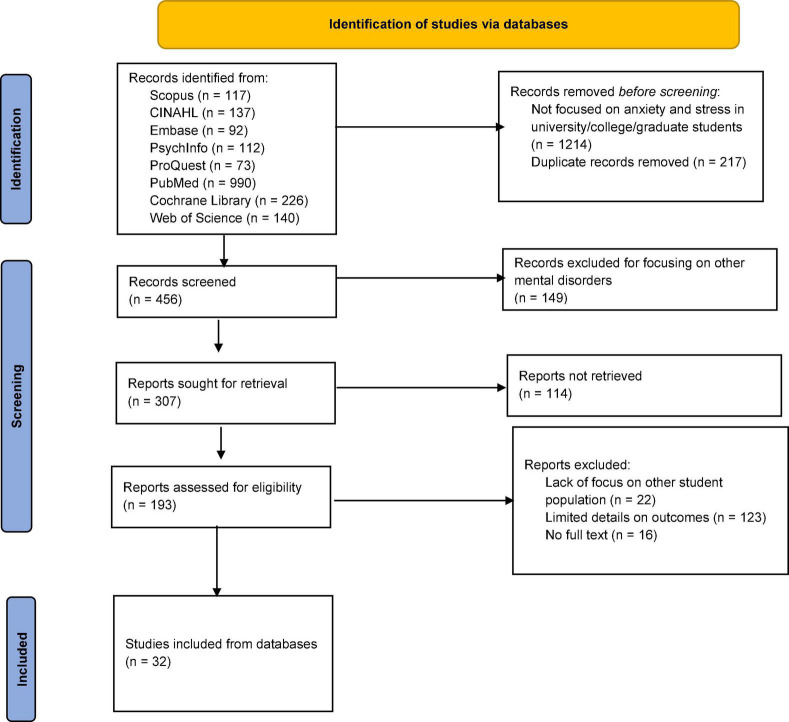
Flowchart.

The keywords used in the search included “psychological stress,” “stress disorders,” “anxiety,” “mental health,” “Coronavirus,” “COVID-19,” “SARS-CoV-2,” “COVID-19 pandemic,” “Russia-Ukraine War,” “students,” “university students,” “college students, “”graduate students.” Keywords were used alone and in Boolean combinations. USA and UK English variations of search terms were used where necessary. The search was initially performed on October 12, 2022, with the final search on October 14, 2022. The search process is illustrated on the PRISMA flowchart in Figure 1.

### Inclusion and exclusion criteria

The title and abstract of each article were screened to determine their relevance to the study’s objectives. The full-text copies were also assessed for eligibility, based on the prescribed criteria. The inclusion criteria were studies (1) reporting stress and/or anxiety related to the COVID-19 pandemic and/or the RUW among graduate, college, or university students; (2) published in the English language; (3) published until October 2022; and (4) having their primary research design as a cross-sectional, longitudinal, or randomized clinical trial.

The studies were excluded if they (1) focused on mental health disorders other than stress and anxiety; (2) were focused on non-university or college students as the population of interest; and (3) were gray literature, abstracts, editorials, case presentations, and letters to the editor.

### Data extraction

Firstly, two reviewers (GAT & RP) independently screened the titles and abstracts of all retrieved studies after duplicates were removed. Secondly, the same reviewers independently read the full-texts of the selected studies in order to evaluate articles for eligibility. More specifically, in order to proceed with more accuracy, the two reviewers first coded 5 papers each (randomly chosen) and then they revised together the data extracted from these ten articles. Discrepancies were resolved through discussion, and little adjustments were made afterward. After this preliminary step, the two authors divided the remaining articles, coded them individually, and checked their reference list for eventual additional resources.

In the datasheet, we extracted the following information from every study: author’s name(s), year of publication, country of origin, study design, sample size, screening tools, primary objective, secondary objective, and main findings.

### Quality appraisal

The study used the Newcastle-Ottawa Scale to assess the quality of the included studies. The adapted Newcastle-Ottawa Scale used by Xiong et al. (16) was applied in this systematic analysis. The studies were assessed on comparability, selections, and outcomes. The three dimensions were assessed using the following seven subcategories: sample size justification, sample representativeness, non-respondents, ascertainment of exposure, design and analysis, outcome assessment, and statistical test. The maximum number of stars that could be awarded to each study was nine, with the comparability, selection, and outcome dimensions having a maximum of two, four, and three stars, respectively.

## Results

### Search results

A total of 1887 articles were identified using the search terms across the different databases and libraries. Out of these, 1214 were excluded for not focusing on university, college, and graduate students. A total of 217 articles were excluded for being duplicates.

The abstracts of 456 remaining articles were retrieved and screened for eligibility. An additional 149 articles were excluded for broadly discussing mental health without focusing on stress and anxiety. Another 161 articles were excluded due to a lack of full text, limited details on anxiety and stress outcomes, and a lack of focus on the target population.

### Characteristics of the included studies

The characteristics of the included studies are summarized in Table 1. The sample size of the included studies ranged from 50 to 29,663 students. A total of 81,395 participants were involved in all 32 studies. In 30 of the 32 studies, female participants comprised over 50% of the sample. Only two of the studies (6.25%) focused on the RUW, while the rest were based on the COVID-19 pandemic. The studies were from nine countries, including the US (*n* = 6), China (*n* = 7), Turkey (*n* = 3), Poland (*n* = 2), Saudi Arabia (*n* = 2), France (*n* = 2), Spain (*n* = 2), Ethiopia (*n* = 1), Czech Republic (*n* = 1), India (*n* = 1), Switzerland (*n* = 2), Ukraine (*n* = 1), and Jordan (*n* = 1). Based on the study design, 31 of the studies were cross-sectional studies, while only one was a longitudinal study. The primary outcomes varied across the included studies. While some only examined either stress or anxiety, some examined both. Only one study measured stress, and the rest included anxiety measures.

**TABLE 1:** Studies’ characteristics.

Lead author year	Country	Study design	Sample size	Sample characteristics	Assessment tool	Primary outcome	Main findings
**COVID19**							
(17)	Saudi Arabia	Cross-sectional study	492	Mean age = 21.77 years old (SD = 2.47); Females = 55.7%	Depression, Anxiety and Stress Scale; Brief-Coping Orientation of Problem Experienced scale	Depression, anxiety, and stress prevalence; Coping strategies	Anxiety and stress prevalence rates were 37.2% and 30.9%, respectively
(18)	USA	Cross-sectional study	50	Participant ages 18 and 21 (56%); Females = 84%; Ethnicities (Asian = 42%, White = 28%, Latinx = 24%, Black = 6%)	Anxiety Symptoms Checklist; College Student Stress Scale	Nature of stress and anxiety; Sources of support	Common symptoms (concentration = 90%, overwhelmed/anxious = 84%, restless = 50%); Sources of Anxiety (academics = 80%, events proceeding as planned = 70%, achieving goals = 50%, finances = 48%); Effects of anxiety (loved one being infected = 84%, one being infected = 70%)
(19)	USA	Longitudinal study	990	Mean age = 21.0 years (SD = 0.54); Females = 61%; White = 58.3%, Black = 8.4%, Asian = 34.2%, Hispanic/Latino = 12.5% and others = 6.2%.	PHQ-4; 14-item Perceived Stress Scale; 20-item UCLA loneliness scale	Anxiety and depression; Stress; Loneliness	Significant increase in anxiety from T1 (*M* = 2.07, SD = 1.64) to T2 (*M* = 2.64, SD = 1.82); For each unit increase in stress at T1, there was a 0.86 unit decrease in the change in anxiety
(20)	China	Cross-sectional study	802	Participants aged 18–26 years; Mean age = 21.31 ± 2.71 years; Females = 602 (62.9%)	Positive and negative emotion scale; Brief COPE prepared by Carver (1997)	Anxiety; fear; sadness; anger	Women experienced significantly higher anxiety than men; Urban participants experienced significantly higher anxiety than rural participants; Closeness to the COVID-19 zone increased the strength of anxiety
(21)	Turkey	Cross-sectional study	646	Participants aged between 16 and 38 (Mean 21.06 ± 2.52); Female = 646 (63.34%)	Turkish version of the Coronavirus Anxiety Scale; Turkish version of the COVID Stress Scale	Stress; Anxiety	13.63% of participants had COVID-Related anxiety; 39.8%, 52.55%, and 7.65% had high, medium, and low stress, respectively; Strong association between stress and anxiety
(22)	France	Cross-sectional study	291	Mean age = 19.07 (SD = 1.7); Females = 73.5%; Social Sciences (16.2%), Health Sciences (26.1%), Technology (32.6%) and Law and Economics (25.1%).	5-point Likert scale on Anxiety, alcohol use, and stress levels	Anxiety; stress; Alcohol use	60.2% reported an increase in anxiety, with rates higher in those confined compared to those who relocated; Stress was higher in those confined (71.6%) compared to those who relocated (50.5%).
(23)	Turkey	Cross-sectional study	411	Participants aged between 18 and 33 years; Mean age = 20.60 ± 1.72; Females = 79.3%; BMI of 65.7% of student = 18.5–24.9/Normal range	Depression, Anxiety, Stress Scale (DASS-42); Dutch Eating Behavior Questionnaire (DEBQ)	Depression; anxiety; Stress	17.8% mild anxiety; 29.7% stress symptoms; Female students more stressed; Stress levels high in students staying with family
(24)	China	Cross-sectional study	29663	Mean age = 21.46 (SD = 2.5); Females = 65.7%	Perceived Stress Scale (PSS); Insomnia Severity Index; Patient Health Questionnaire 9 (PHQ-9)	Stress; Insomnia; Depression	Perceived stress was significantly associated with depression; Insomnia mediated the association between perceived stress
(25)	Spain	Cross-sectional study	2530	Participant ages ranged between 18 and 70 years; Median age = 27.9 years (S.D. = 12.4); Females = 66.1%	Depression, Anxiety, Stress Scale (DASS-21)	Depression; anxiety; stress	Moderate-severe anxiety and stress were 21.34% and 28.14%, respectively; 50.43% reported a significant impact of COVID-19
(26)	USA	Cross-sectional study	2364	Participant ages ranged between 18 and 70 years; Mean age = 25.8 (SD = 8.94); Females = 71.7%	PACT scale; Generalized Anxiety Disorder-7 (GAD-7); COVID-19Related Stress scale	Stress; Anxiety	Trauma-focused coping and forward-focused coping modify the relationship between COVID-19-related anxiety and stress
(27)	Ethiopia	Cross-sectional study	423	Mean age = 22.96 years (range: 18–34); Males = 64.3%	DASS-21	Depression; anxiety; stress	Prevalence of anxiety and stress is 52% and 28.6%, respectively; Anxiety is higher in female, young, non-health department students who do not think that COVID-19 is preventable and do not read COVID-19 prevention materials; Stress higher in female students, with confirmed COVID-19 cases near them, without access to reading material, and with no knowledge of the preventable nature of COVID-19
(14)	USA	Cross-sectional study	195	Mean age = 20.7 (SD = 1.7) years; Females = 57%; 70% in Junior and Senior years	PSS	Stress; Anxiety	Moderate perceived stress (18.8, SD = 4.9); Stress and anxiety increased in 71% during the pandemic; 5% of those with increased stress and anxiety received counseling; 54% of participants had a negative impact of COVID-19 on academics, health, and lifestyle
(28)	USA	Cross-sectional study	2031	Participant ages ranged between 18 and 75 years; Mean age = 22.88 (SD = 5.52); Females = 61.64%	PHQ-9; GAD-7; multiple-choice and open-ended questions regarding stressors and coping mechanisms	Depression; Anxiety; Stress	38.48% had moderate-severe anxiety; 71.26% of participants had increased stress and anxiety levels; 43.25% could cope with stress
(29)	China	Cross-sectional study	3611	Participants aged 18–24 years; Male: Female = 1:1.48;	Self-Rating Anxiety Scale (SAS)	Anxiety	SAS score during COVID-19 higher than the national average; Anxiety higher in females
(30)	Turkey	Cross-sectional study	358	Participants aged 19–40 years; Mean age = 23 years; Females = 55.87%;	GAD-7; PHQ-8; PSS-10; Satisfaction with Life Scale (SWLS)	Anxiety; Stress	The prevalence of anxiety was 52%; Females had higher stress than males; Anxiety and physical inactivity predicted high perceived stress
(31)	India	Cross-sectional study	209	Mean age = 20.33 years (SD = 2.0); Females = 87.56%;	HAM; GAD-7	Anxiety	The prevalence of anxiety was 53.87%; 47.85% = Mild anxiety, 23.36% = Moderate anxiety; 14.35% = moderately severe anxiety, and 1.44% = severe anxiety; Younger (< 20) and female students were more anxious
(32)	Spain	Cross-sectional study	198		GAD-7	Anxiety	Anxiety prevalence was 88.9%
(33)	Egypt	Cross-sectional study	1335	Participants aged 21–22 years (54.8% of sample); Females = 61.8%;	DASS-21	Anxiety; Stress	The prevalence of anxiety was 53.69% and that of stress was 47.8%
(34)	China	Cross-sectional study	740	Medical = 75.81%, Non-medical = 24.19%; Female = 61.68%;	SAS	Anxiety	The prevalence of anxiety was 18.78%; the risk was higher among females (2.164 times higher); knowledge and attitude are protective factors
(35)	China	Cross-sectional study	1396	Mean age = 20.68 years (SD = 1.84); Females = 36.9%	SAS; Self-Rating Depression Scale	Anxiety	Anxiety prevalence was 31%; High physical activity related to low anxiety
(36)	Switzerland	Cross-sectional study	557	Females = 63.8%	PHQ-4	Anxiety	Anxiety prevalence was 85.8%
(37)	Ukraine	Cross-sectional study	1512	Mean age = 20.06 years (SD = 3.05); Females = 69%	GAD-7; PHQ-9	Anxiety	The prevalence of anxiety was 59.13%
(38)	Poland	Cross-sectional study	914	Participants aged 18–40 years; Mean age = 23.04 years (SD = 2.6); Females = 43.11%;	GAD-7; PSS; SWLS; General Self-Rated Health; Coping Inventory for Stressful Situations	Anxiety; Stress; Coping	The prevalence of anxiety was 65% and that of stress was 56%;
(39)	Jordan	Cross-sectional study	1165	Females = 53.8%;	GAD-7; PHQ-9	Anxiety	The prevalence of anxiety was 21.5%
(40)	USA	Cross-sectional study	2282	66% aged 18–24 years; Females = 57.9%	PHQ-4	Anxiety	The prevalence of anxiety was 43.2%
(41)	Switzerland	Cross-sectional study	2437	Females = 70%; Median age = 25 years (23–28)	GAD-7	Anxiety; Stress	The prevalence of anxiety was 61.4%
(42)	France	Cross-sectional study	8004	Medical = 8.35, Non-medical = 91.65%; Age [Medical = 21.11 (3.65); Non-medical = 21.61 (4.25)]	GAD-7; PHQ-9; (Impact of Event Scale-Revised, IES-R)	Anxiety	The prevalence of anxiety was 39.19%; Anxiety and distress were higher in medical students
(43)	Saudi Arabia	Cross-sectional study	400	Age range = 19–25 years; Females = 75.2%	SAS	Anxiety; Coping strategies	The prevalence of anxiety was 34.99%
(44)	China	Cross-sectional study	11787	Mean age = 20.45 (SD = 1.76); Females = 57.11%	GAD-7	Anxiety	The prevalence of anxiety was 17.8%
(45)	China	Cross-sectional study	1912	Mean age = 20.28 years (SD = 2.10, Median = 20, Range = [18, 49]); Female = 69.77%	GAD-7	Anxiety; Stress	The prevalence of anxiety was 34.73%; Mindfulness and social support are protective factors against anxiety; Financial stress significantly predicted traumatic stress
**RUW**							
(3)	Czech Republic	Cross-sectional study	591	56.7% of the participants aged 22 years and below; Females = 67.7%	PHQ-9; GAD-7	Depression; anxiety	22.3% and 13.7% of participants had moderate and severe symptoms; Females had higher anxiety than males. Students from urban areas and cities with large populations had more anxiety
(46)	Poland	Cross-sectional study	510	Median age = 21; Females = 65%; 61% Christians and 16% atheists	State-Trait Anxiety Inventory (STAI)	Anxiety	Anxiety was higher in females; year 1 students had higher anxiety overall than year 5 students; Anxiety as a trait was higher in year 6 students

### Measurement tools

The instruments used to measure anxiety and stress included the Depression, Anxiety and Stress Scale; Patient Health Questionnaire 4 (PHQ-4); 14-item Perceived Stress Scale; Positive and negative emotion scale; Coronavirus Anxiety Scale; Coronavirus Stress Scale; Perceived Stress Scale (PSS); Generalized Anxiety Disorder-7; Impact of Event Scale-Revised; Self-Rating Anxiety Scale (SAS); Coping Inventory for Stressful Situations; and State-Trait Anxiety Inventory. Five reported interventions and coping mechanisms for stress and anxiety were observed in the postsecondary student samples.

### Quality appraisal

The outcome of the study quality appraisal is presented in Table 2. Overall, the quality of the included studies was 6.22, above the cut-off point of 4.5. Among the studies, 4 had five stars, 14 had six stars, 10 had seven stars, 2 had eight stars, and 1 study had nine stars. Overall, all the included studies were considered high quality as they were above the cut-off point of the NOC assessment scale.

**TABLE 2:** Outcome of the studies quality appraisal.

Lead author year	Selection				Comparability	Outcome		Total score
	Representativeness of sample	Sample size	Non-respondents	Ascertainment of exposure		Assessment of outcome	Statistical test	
**COVID19**								
(17)	*			*	**	*	*	6
(18)	*			*	**	*	*	6
(19)	*		*	*	**	*	*	7
(20)	*		*	*		*	*	5
(21)	*			*	**	*	*	6
(22)	*			*	*	*	*	5
(23)	*			*	**	*	*	6
(24)	*	*	*	*	**	*	*	9
(25)	*			*	**	*	*	6
(26)	*		*	*	*	*	*	6
(27)	*			*	**	*	*	6
(14)	*			*	**	*	*	6
(29)	*		*	*	**	*	*	7
(30)	*			*	*	*	*	5
(31)	*		*	*	**	*	*	7
(32)	*		*	*	**	*	*	7
(33)	*		*	*	**	*	*	7
(34)	*			*	**	*	*	6
(35)	*		*	*	*	*	*	7
(36)	*		*	*	*	*	*	7
(37)	*			*	**	*	*	6
(38)	*			*	*	*	*	5
(39)	*		*	*	**	*	*	7
(40)	*			*	**	*	*	6
(41)	*		*	*	**	*	*	7
(42)	*	*	*	*	**	*	*	8
(43)	*			*	**	*	*	6
(44)	*	*	*	*	**	*	*	8
(45)	*			*	**	*	*	6
**RUW**								
(3)	*		*	*	**	*	*	7
(46)	*			*	**	*	*	6

* and ** represent a 1-point value, indicating the presence or absence of each criterion, per study.

### Anxiety and stress prevalence and predictors

The prevalence of stress and anxiety was measured in 31 out of the 32 studies. The prevalence of anxiety was as high as 88.9% (32) and as low as 13.63% (21). Some of the studies reported significantly higher rates of anxiety of over 70% among the respondents (14, 28, 32, 36). A majority of the studies reported prevalence rates above 50%. Four studies assessed anxiety’s prevalence based on severity (3, 25, 28, 31). Wang and Zhao (28) found that moderate to severe anxiety prevalence was 38.48%, while Riad et al. (3) reported that the prevalence rate was 36%. Odriozola-González et al. (25) found that moderate and severe stress prevalence was 21.34% and 28.14%, respectively, with Biswas and Biswas (31) reporting the same as 23.36% and 1.44%, respectively. Wang et al. (29) found that the university anxiety rate reported during the pandemic was higher than the national average.

The predictors of stress and anxiety included young age, gender being female, STEM courses, loneliness, low academic level in school, urban lockdown, confinement, having a preexisting disease, having relatives or friends infected with COVID-19, and proximity to a COVID-19 zone (18, 20, 22, 25, 27, 29, 31, 34).

Gender was identified as an important factor in determining the prevalence and severity of anxiety. Female students were identified to have higher anxiety levels due to the COVID-19 pandemic compared to their male counterparts (20, 25, 27, 29, 31, 34). Anxiety was also higher in female students compared to the male students in the RUW studies (3, 46).

The stress prevalence rate ranged from 56 (38) to 28.14% (25). Most of the studies reported stress prevalence between 20 and 40%. Among the studies reporting stress, Durbas et al. (21) found that medium stress (53.55%) was more prevalent than high (39.8%) and low stress (7.65%). The predictors of stress included gender being female, living with family, living in a household with many people, being confined rather than having the freedom to relocate, proximity to confirmed cases of COVID-19, lack of access to materials on COVID-19, preexisting mental disorders, and lack of knowledge on the preventable nature of COVID-19 (17, 19, 22–24, 27). Gender also predicted stress, with female students being more stressed than male students (23, 27, 30, 38). Overall, the prevalence of stress was generally lower than the prevalence of anxiety (27, 33, 37, 38). Uğurlu et al. (23) and Odriozola-González et al. (25) reported higher rates of stress than that of anxiety.

### Symptoms and risk factors of stress and anxiety

The symptoms of anxiety and their associated risk factors were examined in 31 of the 32 studies. The symptoms of anxiety identified in the included studies included loss of concentration, feeling overwhelmed, and restlessness (18, 19, 25, 27, 38, 42). The sources of anxiety among the university students identified in the study included academics, postponement of graduation, cancelation or disruption of planned events, inability to achieve goals, and finances (18, 19, 27, 40). The effects of anxiety identified in the studies included the fear of being infected and the fear of a loved one being infected (18, 27, 38, 42). The courses students took in school were a risk factor associated with stress. The students with courses from the non-health department had lower anxiety than their peers from the non, health, art, and liberal courses (27). Odriozola-González et al. (25) found that the students who took arts, humanities, social sciences, and law courses had significantly high moderate and severe stress and significantly lower subclinical stress bases of the IES categories compared to those in the engineering and architecture, science, and health science courses.

### Coping and protective factors

Researchers also examined students’ coping styles and strategies during the pandemic (20, 26, 28, 38, 43). Rudenstine et al. (26) examined trauma-focused and forward-focused coping styles and their impacts on stress and anxiety. Using a low-income student sample, the researchers found that both forms of coping modified the relationship between COVID-related stress and anxiety symptoms. Rogowska et al. (38) examined task-oriented, emotion-oriented, and avoidance-oriented coping styles and their impacts on stress and anxiety. Emotion-oriented and avoidance-oriented coping styles were associated with high anxiety levels, with no correlation identified between anxiety and the use of task-oriented coping strategies. The students who used task-oriented coping styles managed to reduce their stress levels while increasing anxiety.

Khoshaim et al. (43) examined four coping strategies and found that avoidance was the most preferred coping strategy, followed by mental disengagement and humanitarian work. The researchers found that support-seeking was the least-used coping strategy, with its use being significantly lower in male students. Huang et al. (20) compared the use and impact of problem-focused and emotion-focused coping strategies among nursing students. They found that problem-coping was the most utilized strategy. The researchers also found that problem-coping led to increased anxiety among the participants. While 71.26% of participants in the study by Wang and Zhao (28) reported increased stress and anxiety, only 43.25% utilized coping mechanisms.

The researchers identified multiple protective factors that reduced stress and anxiety. The protective factors identified in the included studies included spirituality/religion, mindfulness, social support, physical activity, and knowledge about infection prevention and treatment (17, 26, 28, 35, 45).

### Association between stress and anxiety

Five of the studies reported the relationship between stress and anxiety (19, 21, 24, 30, 38). Durbas et al. (21) found a strong association between stress and anxiety, while Aslan et al. (30) found that the existence of anxiety and physical inactivity predicted stress among students during the COVID-19 pandemic. Additionally, other mental disorders were examined to understand their relationship with stress and anxiety. Liu et al. (24) found a significant association between stress and depression. Additionally, insomnia was found to mediate between perceived stress and perceived anxiety. The longitudinal study by Haikalis et al. (19) identified an inverse relationship between changes in anxiety and stress. The researcher reported a 0.86 unit decrease in anxiety for each unit increase in stress. Finally, Rogowska et al. (38) found high anxiety levels related to high stress levels.

## Discussion

Our review examined the mental health outcomes, protective factors, and predictive factors during the COVID-19 pandemic and the RUW. Natural disasters and man-made events can disrupt college students’ daily lives and increase their stress and anxiety levels. Generally, the prevalence of stress and anxiety increased during the pandemic and the war. Gender differences were observed in the prevalence of stress and anxiety and the coping mechanisms used by the students. The female students also tended to have high levels and severity of stress and anxiety than male students. While the male students utilized problem-focused coping strategies, the female students used support-focused coping mechanisms.

Haikalis et al. (19) study was the only longitudinal study in our review that compared patients’ anxiety in an ongoing pre-pandemic study with the outcomes during the pandemic. The PSS and PHQ instruments showed that students’ stress and anxiety increased with the campus being closed compared to how they were in the pre-pandemic period. The increased anxiety and stress levels in the pandemic and the war reported by the studies are attributed to multiple factors. First, the uncertainties and instabilities created by the pandemic and the war explain the rise in the stress and anxiety of the surveyed students.

The findings on the moderating role of the courses taken by the students were mixed. While some researchers (27) found that the students in the STEM courses, such as health and medicine, had lower perceived stress and anxiety levels than those in non-STEM courses, such as arts, Essadek et al. (42) found that the medical students had higher anxiety than non-medical students.

The stages in the pandemic and RUW are key variables that potentially explain variances in stress and anxiety prevalence and differences in the coping mechanisms used. Khoshaim et al. (43) reported that avoidance was the most used strategy, while Rogowska reported significant use of task-oriented coping strategies. Given the ineffective nature of these strategies in reducing anxiety over the long term, their use might be important in only the early stages when the studies were conducted. Nevertheless, the findings demonstrate the value of using diverse coping strategies and matching them to the stage or duration of a pandemic or war to reduce anxiety.

The findings on the relationship between stress and anxiety during the pandemic and RUW are important in informing the decisions on implementing coping strategies. With Rogowska et al. (38) reporting using task-oriented coping to effectively manage stressful events leading to increased anxiety, the findings support the current knowledge on problem-focused coping. On the other hand, Kasi et al. (47) found that while problem-focused coping is common among people experiencing anxiety, it does not always guarantee the expected results. The uncertainty presented by the COVID-19 pandemic and the RUW implies that problem-focused coping is insufficient as one gets increasingly frustrated by developments outside their control.

Khoshaim et al. (43) and Huang et al. (20) found that while problem-focused coping is common among people experiencing anxiety, it does not always guarantee the expected results. The uncertainty presented by COVID-19 and the RUW implies that problem-focused coping is insufficient as one is increasingly frustrated by new developments. The findings have practical value that can be applied in higher learning institutions. The majority of the respondents reported poor or limited coping strategies and limited psychological support as predictive factors for stress and anxiety (33). Of the 71% of students who reported that they had stress and anxiety, Son et al. (14) reported that only 5% received counseling. This emphasizes the need for building coping mechanisms for students in the post-pandemic period. Higher learning institutions have an opportunity to provide psychological services during pandemics such as the COVID-19 pandemic and crises such as the RUW to mitigate their emotional impact.

Overall, the prevalence of stress and anxiety were significantly higher in female students than in male students. This points out the need to view female students as a vulnerable group. However, with Khoshaim et al. (43) reporting that female students are more likely to seek support as a coping mechanism while male students use avoidance and mental disengagement, the implications on both genders over the long term are likely to differ. The ineffective problem-focused coping strategies that male students prefer might be counterproductive, as crises or disasters last for longer than initially expected. Huang et al. (20) proposed that longer studies are needed to determine if the impact of problem-focused coping strategies on anxiety persists. Besides females, other studies have established that students of minority sexual orientation also experienced higher stress and anxiety during the pandemic (48). These findings emphasize the need to segment students based on risk and prioritize the vulnerable ones during pandemics and crises to improve outcomes.

The findings also emphasize the value of a multipronged approach in mitigating the negative effects of pandemics and wars on postsecondary students. The presence of mental health services that can be accessed beyond the physical premises of higher-learning institutions is one of the key adjustments that universities can make. According to Husky et al. (22), counseling is important in ensuring that students can manage their psychological outcomes when events such as graduations are postponed. Other than telemental health services, the financial, housing, and food insecurities that increase stress and anxiety during pandemics and wars must be addressed. Additionally, institutions transitioning to virtual learning need to offer orientation and training. By focusing on low-income students, Rudenstine et al. (26) provided a unique perspective on how interventions can be adjusted to meet the needs of a specific group. The study is significant as it emphasizes the need for coping mechanisms to be structured to meet the various needs of different groups, despite them experiencing the COVID-19 pandemic and the RUW simultaneously. Learning institutions and public health organizations need to understand that a one-size-fits-all approach cannot effectively address the diverse student populations experiencing stress and anxiety during a pandemic.

Universities need to partner with other public health institutions to inform learners about infectious diseases. Jia et al. (34) and Simegn et al. (27) found that the level of stress and anxiety decreased when the students were informed about COVID-19 infection and prevention. The active dissemination spread of misinformation is an important protective factor that can be used to mitigate stress and anxiety. The awareness campaign should be part of a comprehensive communication plan. While college students can autonomously acquire knowledge on the pandemic or the RUW, an authoritative institution needs to disseminate the information to reduce the chances of the spread of misinformation.

Beyond the psychological impact, the workload or emphasis on academic performance is another area to be reexamined during pandemics and crises to improve student outcomes. Previous studies have found that academic performance is a key source of stress and anxiety (14, 18). Workload management and timely communication with students during crises might help mitigate or reduce the severity of their stress and anxiety.

### Strengths

To the best of our knowledge, this is the first systematic review of the literature on the prevalence and impact of the COVID-19 pandemic and the RUW on stress and anxiety in postsecondary students. The focus on postsecondary students is important as it helps understand factors such as the resilience of the group facing the most significant natural disaster of their lives and experiencing a war at the brink of threatening global security. The rigorous inclusion criteria ensured that the included studies were focused on anxiety and stress among postsecondary students, thus increasing the understanding of the dynamics of the two mental disorders in the group during the pandemic and the RUW. The selection process was also specific to the design of the included articles. As proven by the NOC article quality analysis, the cross-sectional and longitudinal studies provided high-quality findings that were utilized to identify useful recommendations and draw important conclusions.

### Limitations and future directions

The lack of a quantitative analysis of the included studies is one of the limitations of this study. This study did not have a meta-analysis component, as the assessment tools varied across the studies, and different study designs were used. The absence of pooling implies that the current study cannot sufficiently explain the prevalence of stress and anxiety variation. Approximately, 97% of the studies were cross-sectional. This implied that the level of anxiety was measured at a single point. The lack of comparison with the pre-pandemic or the post-pandemic period reduces the value of the findings. The studies also examined the anxiety and stress of the students during different periods of the pandemic. This explains the significant variance in prevalence rates, with some studies reporting over 95% prevalence while others reporting less than 10%. No studies examined the joint effects of the pandemic and the RUW. No longitudinal studies examined the change in stress and anxiety attributed to each event.

Future studies need to consider the impact of simultaneously experiencing the pandemic and the war. Longitudinal studies comparing the differences in stress and anxiety in the post-pandemic period are necessary for understanding the effects of the pandemic’s long-term stress and anxiety. Future longitudinal studies will also help in understanding the impact of stress and anxiety on outcomes such as academic performance and the students’ overall well-being, thus providing opportunities for targeted interventions. The outbreak of the RUW toward the end of February 2022 implied that few studies focused on the mental disorders and targeted population for the present study. This led to identifying a few studies that fit the inclusion criteria. A larger pool of studies on student stress and anxiety that focus on the RUW will be important in improving the quality of future systematic reviews.

In addition to this, it could be of interest deepen the neurobiological mechanisms that underly stress and anxiety in relation to facing a crisis. The biological perturbations observed in patients that have faced trauma are numerous, and reflect a dysregulation of multiple stress-mediating systems: a lack of baseline Cortisol at the time of a psychological trauma resulting in enhanced and prolonged stress response, inadequate regulatory effects of GABA, and serotonin, altered norpinephrine and stress hormone activity, elevated noradrenergic activity and relative hypocortisolism, abnormally functioning hippocampus, exaggerated amygdala responses and disrupted prefrontal cortical function (49). Future studies should explore possible differences in these neurobiological mechanisms in relation to different types of crises such as a pandemic or a war in order to better inform mental health interventions aimed at supporting traumatized students.

## Conclusion

The studies included in the review showed that stress and anxiety increased during the pandemic and war, with gender and uncertainty playing a critical role in it. The studies provided insights into the widespread use of problem-focused and task-focused coping strategies, despite their impact on increasing stress and anxiety. Higher learning institutions have an opportunity to provide interventions for students going through the effects of the COVID-19 pandemic, the RUW, and other future crises. Longitudinal studies provide an opportunity for understanding students’ long-term stress and anxiety outcomes associated with the COVID-19 pandemic and the RUW and the impact of different coping strategies.

## Author contributions

PL: introduction. GM: methodology. GT: data and conclusion. All authors contributed to the article and approved the submitted version.
